# SplicePlot: a utility for visualizing splicing quantitative trait loci

**DOI:** 10.1093/bioinformatics/btt733

**Published:** 2013-12-19

**Authors:** Eric Wu, Tracy Nance, Stephen B. Montgomery

**Affiliations:** Department of Pathology, Stanford University School of Medicine, Stanford, CA 94305-5324, USA

## Abstract

**Summary**: RNA sequencing has provided unprecedented resolution of alternative splicing and splicing quantitative trait loci (sQTL). However, there are few tools available for visualizing the genotype-dependent effects of splicing at a population level. SplicePlot is a simple command line utility that produces intuitive visualization of sQTLs and their effects. SplicePlot takes mapped RNA sequencing reads in BAM format and genotype data in VCF format as input and outputs publication-quality Sashimi plots, hive plots and structure plots, enabling better investigation and understanding of the role of genetics on alternative splicing and transcript structure.

**Availability and implementation:** Source code and detailed documentation are available at http://montgomerylab.stanford.edu/spliceplot/index.html under Resources and at Github. SplicePlot is implemented in Python and is supported on Linux and Mac OS. A VirtualBox virtual machine running Ubuntu with SplicePlot already installed is also available.

**Contact:** wu.eric.g@gmail.com or smontgom@stanford.edu

## 1 INTRODUCTION

Alternative splicing is a post-transcriptional process in which exonic regions of the pre-mRNA are spliced together and intronic regions are removed. Because numerous combinations of exons can be spliced together, splicing contributes significantly to the diversity of the transcriptome and the resultant proteome. Further adding to this diversity, genetic variation in cis-acting splice site regulators and trans-acting splicing factors can affect patterns of splicing between individuals and potentially manifest as phenotypic differences (Cooper *et al.*, 2009). Here, the study of splicing quantitative trait loci (sQTLs) through the statistical testing of genetic association with changes in alternative splicing proves useful in identifying functional effects that may contribute to the etiology of various genetic diseases.

The invention of RNA sequencing (RNA-seq) provides high resolution, single base characterization and quantification of the transcriptome, providing enhanced resolution of patterns of alternative splicing. Specifically, using RNA-seq, the usage of exons and splice junctions can be precisely quantified, enabling the comparison of splicing between individuals in a population. Indeed, several studies have reported on extensive diversity of sQTL detectable from RNA-seq data (Montgomery *et al.*, 2010; Pickrell *et al.*, 2010).

However, currently there are few (if any) existing tools that provide convenient and effective visualizations of sQTL and their effects in RNA-seq data. Existing tools, such as the Sashimi plot provided within the MISO package (Katz *et al.*, 2013), are capable of showing differential splicing between individual samples, but not between genotypes (a three-way comparison). Because the study of sQTLs is, by nature, a study of individuals within a population, effective sQTL visualization requires tools that integrate population-scale RNA-seq datasets and genotype information to visualize the effect of genetic variation on alternative splicing.

Here, we present SplicePlot, a command-line utility for visualizing the effects of genetic variants on alternative splicing and transcript structure. It produces hive plots (Krzywinski *et al.*, 2011) and structure plots, as novel ways of visualizing and comparing patterns of splicing as measured by splice junction-spanning reads, by genotype. It also modifies and extends the Sashimi plot to support the comparison of splicing and transcript structure between genotypic groups. Such visualizations take advantage of the high precision quantification of RNA-seq and enable a better understanding of the genetic effects behind alternative splicing in RNA-seq data.

## 2 USAGE AND IMPLEMENTATION

SplicePlot requires three main inputs: (i) mapped RNA-seq reads in BAM format, created by a read mapper capable of mapping junction-spanning reads; (ii) genotypes for each individual in VCF format; and (iii) an annotation of known exons in GTF format, which can be downloaded from online databases like UCSC or Ensembl and processed using a provided script. The user specifies the variant position and splice junction at the command line. SplicePlot produces publication-quality images in SVG format. These images are highly customizable, as plotting parameters like colors, dimensions and font sizes can be specified using a settings file.

Using the supplied annotation of known exons, SplicePlot determines the genomic window of interest and then determines the read depth and the number of junction-spanning reads in the window for each individual. To produce hive plots and structure plots, splicing ratios for each individual and junction are calculated using the following formula:

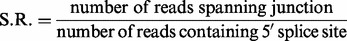



Intuitively, the splicing ratio for a 5′ splice site represents the proportion of junction-spanning reads containing the five splice site that also contains a particular 3′ splice site. A similar formula for 3′ splicing ratios can also be defined. SplicePlot also calculates the average read depth and the average number of junction-spanning reads for groups stratified by a genotype at a user-specified locus, for use in drawing modified Sashimi plots.

SplicePlot is implemented in Python using the pysam module, a wrapper for SAMtools (Li *et al.*, 2009). Plotting is fast and memory-efficient, as retrievals from VCF and GTF files are done with Tabix (Li *et al.*, 2009).

## 3 RESULTS

### 3.1 Hive plots

**Fig. 1.**
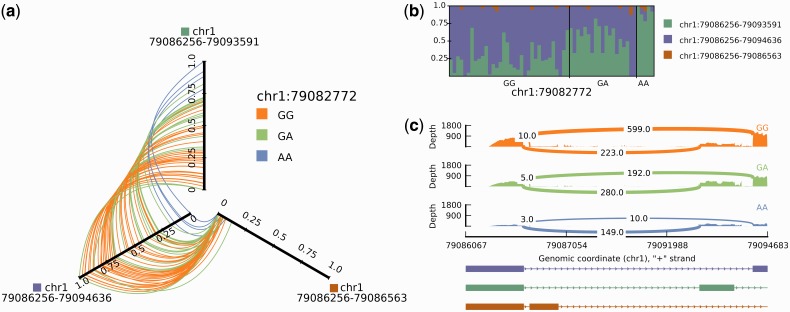
Example plots illustrating the same sQTL in 59 individuals. (**a**) A hive plot. (**b**) A structure plot. (**c**) A Sashimi plot

A hive plot is a radial plot that enables the comparison of splicing ratios between individuals and between genotype groups. Each of the axes corresponds to a splice junction with a fixed donor (or acceptor) location. Individuals are represented by curved paths that go around the plot. The paths are color-coded by the individual’s genotype at the user-specified locus. The radial distances of the points of intersection between the paths and the axes are proportional to the splicing ratio for the corresponding splice junction in the individual. The approximate strength of the sQTL can be estimated by looking at the color patterns in the plot. A sample hive plot is shown in Figure 1a.

### 3.2 Structure plots

Structure plots [named after popular visualizations used by the program STRUCTURE, (Hubisz *et al.*, 2009)] provide another way of visualizing sQTLs in populations, while showing splicing ratios at the individual level. In a structure plot, each set of stacked bars represents the splicing ratios for each individual. The bars are color-coded by junction, and the height of each bar represents the magnitude of the splicing ratio. In addition, the arrangement of stacked bars clearly shows that the sum of the splicing ratios in each individual must total 1. Individuals are grouped spatially by their genotype at the specified locus to enable the viewer to effectively compare the similarities and differences in splice junction usage by genotype. A sample structure plot is shown in Figure 1b.

### 3.3 Modified Sashimi plots

SplicePlot modifies the Sashimi plot from MISO to enable the effective visual comparison of alternative splicing and transcript structure across genotypic categories within populations. The plots allow the average read depth and the average number of junction-spanning reads to be compared between genotype groups, providing insight into the ways sQTL affects the quantity and structure of isoforms. A sample Sashimi plot is shown in Figure 1c.

## 4 CONCLUSION

SplicePlot is an analytic tool for summarizing read data and generating intuitive, publication-quality figures for visualizing sQTL. It draws hive plots, structure plots and Sashimi plots to enable the effective comparison of alternative splicing and transcript structure. We have further provided an Ubuntu virtual machine containing the Montgomery *et al.* (2010) dataset with SplicePlot. This virtual machine can be downloaded and installed to allow users to investigate loci of interest, thereby providing new opportunity for the research community to investigate the impact of any genetic variant on splicing.

*Funding*: Edward Mallinckrodt Jr. Foundation.

*Conflict of Interest*: none declared.
